# Insights into Hydrogen Diffusion Characteristics and Interactions with Vacancy in Fe Crystal Lattices from First-Principles Calculations

**DOI:** 10.3390/ma19061175

**Published:** 2026-03-17

**Authors:** Yi Feng, Maoqing He, Guangjie Huang, Wenjuan Zhao, Zhihui Cai, Deliang Zhang, Jianing Bao

**Affiliations:** 1 College of Materials Science & Engineering, Chongqing University, Chongqing 400044, China; 2 China Automotive Engineering Research and Development Co., Ltd., Chongqing 401122, China; 3 School of Mechanical Engineering and Mechanics, Xiangtan University, Xiangtan 411105, China; 4 Hunan Rare Earth Metal Materials Research Institute Co., Ltd., Changsha 410126, China; 5 State Key Laboratory of Advanced Stainless Steel, Taiyuan University of Science and Technology, Taiyuan 030024, China

**Keywords:** Fe crystal lattice, first-principles, hydrogen diffusion, vacancy

## Abstract

Hydrogen embrittlement is defined as the phenomenon wherein materials undergo sudden degradation in mechanical properties due to the ingress of hydrogen atoms, and its occurrence is closely linked to hydrogen diffusion behavior. Here, first-principles calculations are employed to systematically investigate the hydrogen diffusion characteristics of both perfect and vacancy-containing α-Fe, γ-Fe, and ε-Fe crystal structures. The dissolution energies of hydrogen atoms in perfect α-Fe, γ-Fe, and ε-Fe crystals were calculated at different interstitial sites and transition states along various pathways. Hydrogen atoms preferentially occupy tetrahedral interstitial sites in α-Fe crystals, with diffusion occurring between two nearest-neighbor tetrahedral interstitial sites. In γ-Fe crystals, hydrogen atoms favor octahedral interstitial sites, diffusing along paths from octahedral sites to tetrahedral sites and then to other octahedral sites. In ε-Fe crystals, hydrogen atoms preferentially occupy octahedral interstitial sites and diffuse along pathways between nearest octahedral interstitial sites. The hydrogen diffusion coefficients calculated based on the Arrhenius equation follow the order α-Fe > γ-Fe > ε-Fe, indicating that hydrogen atoms diffuse most readily in α-Fe crystals. Notably, examination of the relationship between the interatomic distance and interaction energy in α-Fe reveals that hydrogen atoms have difficulty aggregating and forming hydrogen molecules within defect-free α-Fe crystals. However, introducing vacancy defects increases the mutual attraction between hydrogen atoms, thereby facilitating hydrogen bubble nucleation. Furthermore, the introduction of vacancy defects in α-Fe, γ-Fe, and ε-Fe alters the preferential occupancy sites and diffusion pathways of hydrogen because of vacancy trapping effects. Compared with diffusion in perfect crystals, hydrogen atoms must overcome substantially higher energy barriers to escape vacancy trapping and diffuse into defect-free lattice regions.

## 1. Introduction

The global transition toward a green economy has accelerated the utilization of hydrogen energy and the development of hydrogen-based technologies, which require an increasing number of steel components to serve in hydrogen-rich environments [1,2]. While hydrogen is widely recognized as an efficient clean energy source, its interaction with steel materials can lead to severe mechanical degradation, including strength reduction, plasticity deterioration, and embrittlement cracking. This phenomenon is collectively termed hydrogen embrittlement. Moreover, as fundamental engineering materials in the era of carbon neutrality, steels increasingly require higher strength and extended service life [3,4]. Unfortunately, this critical enhancement of mechanical properties is associated with increased susceptibility to hydrogen embrittlement—a detrimental trade-off that significantly constrains their practical applications and poses substantial challenges for material design [5]. Extensive research efforts have yielded multiple theoretical frameworks, including hydrogen pressure theory [6], the hydrogen-enhanced decohesion mechanism (HEDE) [7], hydrogen-enhanced localized plasticity (HELP) [8], and hydrogen-enhanced strain-induced vacancy formation (HESIV) [9] mechanisms to explain hydrogen embrittlement phenomena. While these theories have advanced the fundamental understanding, each exhibits certain limitations in comprehensively accounting for all observed hydrogen-induced failure modes. Qiao et al. [10] reported that hydrogen atoms can diffuse into stainless steel and accumulate at critical microstructural features such as crack tips and lattice defects, thereby governing crack initiation, propagation, and growth. These findings confirm that hydrogen embrittlement fundamentally originates from the complex interplay between diffusing hydrogen atoms and the host metallic matrix. This process triggers crack nucleation and extension, ultimately leading to catastrophic fracture. Consequently, both the nucleation and the growth of hydrogen-induced cracks are closely related to the diffusion process of hydrogen atoms. In-depth research on the interaction between hydrogen and the metal matrix, as well as the hydrogen diffusion behavior, is critically important for understanding and ultimately resolving hydrogen embrittlement issues.

Park et al. [11] experimentally demonstrated that acicular ferrite, bainite, and martensite phases significantly influence both hydrogen diffusion kinetics and hydrogen-induced cracking susceptibility in pipeline steels. This phase-dependent behavior originates from the polymorphic nature of iron, which is present in body-centered cubic (BCC), face-centered cubic (FCC), and hexagonal close-packed (HCP) crystal structures [12]. Each structure exhibits distinct hydrogen diffusion mechanisms. A systematic understanding of hydrogen diffusion in iron therefore necessitates comparative studies of hydrogen diffusion behavior across these different crystalline structures. Owing to limitations in current experimental techniques, conventional methods cannot accurately observe hydrogen diffusion mechanisms at the atomic scale. However, with rapid advances in computational technology and theoretical methodologies, numerical simulation has become an indispensable tool for investigating hydrogen embrittlement-related phenomena [13]. Sanchez et al. [14] employed molecular dynamics simulations to study hydrogen diffusion in α-Fe; however, they did not perform transition state analysis at the microscopic level. The diffusion energy barriers obtained from transition state searches can indirectly reflect the hydrogen diffusion coefficients, which are critical parameters for determining the hydrogen transport rates in materials and directly influence the susceptibility to hydrogen embrittlement. Therefore, in this study, diffusion coefficients are calculated on the basis of energy barrier analysis, providing a quantitative framework for understanding hydrogen diffusion behavior in iron. Jiang et al. [15] utilized first-principles calculations to demonstrate that hydrogen atoms in BCC Fe diffuse along a curved trajectory between tetrahedral interstitial sites rather than migrating along straight paths. Furthermore, Lu et al. [16] employed numerical simulations to investigate hydrogen diffusion in vacancy-containing α-Fe. Their results demonstrated that the binding energy of hydrogen in defect-free lattice sites is significantly lower than that at vacancy sites, indicating that vacancies act as effective traps that impede hydrogen diffusion. These findings clearly reveal the strong interaction between diffusing hydrogen atoms and crystalline defects (vacancies), which thereby affects the hydrogen embrittlement sensitivity of the material.

In this study, first-principles calculations based on density functional theory (DFT) calculations are employed to systematically investigate the hydrogen occupation preferences and diffusion mechanisms in three iron allotropes (α-Fe, γ-Fe, and ε-Fe). Comprehensive transition state analysis of various diffusion pathways enables the calculation of diffusion coefficients through the Arrhenius equation, providing a quantitative comparison of the hydrogen diffusion rate across different crystalline structures. Furthermore, two critical aspects governing hydrogen behavior are examined: interatomic interactions between hydrogen atoms in α-Fe lattices and the significant influence of vacancy defects on hydrogen transport properties in all three iron phases. These investigations yield a theoretical foundation for atomic-scale hydrogen diffusion in iron-based materials.

## 2. Modeling and Simulation

### 2.1. First-Principles Simulations

First-principles calculation is a quantum mechanics-based method for solving the Schrödinger equation that does not rely on experimental data or empirical parameters [17]. By employing this method, it is possible to analyze the crystal structure, electronic structure, and other properties of materials from a microscopic perspective. This thereby provides reliable theoretical support for elucidating the underlying principles of experimental phenomena. The calculations in this study were performed within the CASTEP package, a first-principles calculation program implementing DFT and the plane-wave pseudopotential technique [18]. The ultrasoft pseudopotential and plane-wave basis set were employed to represent the valence electron wavefunctions, while the exchange-correlation energy was described using the Perdew–Burke–Ernzerhof (PBE) function within the generalized gradient approximation (GGA) [19,20]. The computational setup incorporated a 10 × 10 × 10 Monkhorst–Pack k-point mesh for Brillouin zone integration, with the plane-wave basis set truncated at 330 eV [21]. Structural optimizations were performed using the Broyden–Fletcher–Goldfarb–Shanno (BFGS) algorithm [22]. To ensure computational accuracy, we implemented stringent convergence criteria: the self-consistent field (SCF) tolerance was set to 1.0 × 10^−6^ eV/atom, while the total energy convergence threshold was maintained at 1.0 × 10^−5^ eV/atom. Additional convergence parameters included the maximum stress (0.05 GPa), maximum force (0.03 eV/Å), and maximum displacement (0.001 Å) per atom. To elucidate the hydrogen diffusion mechanism, this work utilizes the complete LST/QST approach within the “TS search” module of CASTEP to identify diffusion pathways and transition states of hydrogen atoms across various unit cells [23].

### 2.2. Computational Model

**Figure 1:**
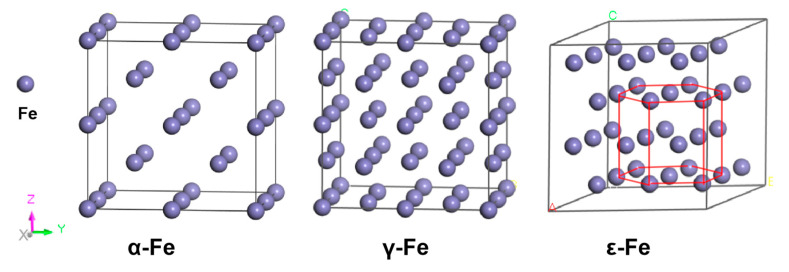
Schematic atomic models of the three Fe crystal configurations.

Fe has three crystalline phases: a body-centered cubic (BCC) α-Fe crystal, a face-centered cubic (FCC) γ-Fe crystal, and a hexagonal close-packed (HCP) ε-Fe crystal [24]. For computational modeling, in this work, 2 × 2 × 2 supercells for both α-Fe and γ-Fe crystals and a 3 × 3 × 2 supercell for the ε-Fe phase were constructed, as shown in Figure 1. The computational framework treated γ-Fe and ε-Fe as nonmagnetic crystals, whereas spin polarization was considered for the ferromagnetic α-Fe crystal. Structural optimizations were performed under periodic boundary conditions for all three crystals. The calculated lattice parameters yielded a = b = c = 2.851 Å for α-Fe, a = b = c = 3.456 Å for γ-Fe, and a = b = 2.46 Å, with c/a = 1.581 for ε-Fe, demonstrating excellent agreement with previously reported experimental and computational studies, as shown in Table 1.

### 2.3. Solution Energy Calculations

The solution energy serves as a quantitative descriptor of the thermodynamic propensity of hydrogen atoms to incorporate into the host lattice, which is formally defined as follows [15,32]:

(1) $$E_{sol}=E(Fe_{n}H)-E(Fe_{n})-\frac{1}{2}E(H_{2})$$

where $E(Fe_{n}H)$ represents the total energy of the supercell system containing one hydrogen atom, $E(Fe_{n})$ represents the total energy of the hydrogen-free supercell system, and $E(H_{2})$ denotes the ground-state energy of H_2_, with a calculated value of −31.675 eV [33]. As evident from Equation (1), a negative solution energy ($E_{sol}$ < 0) corresponds to an exothermic solution process, indicating thermodynamically favorable hydrogen incorporation into the metallic matrix. Conversely, positive solution energies ($E_{sol}$ > 0) characterize endothermic reactions where spontaneous solution becomes energetically unfavorable.

### 2.4. Calculation Method for the Diffusion Coefficient

The relationship between the hydrogen diffusion coefficient and temperature in the Fe lattice can be described by the Arrhenius equation as follows:

(2) $$D=D_{0}\exp\left(-\frac{E_{a}}{k_{B}T}\right)$$

where D and $D_{0}$ represent the diffusion coefficient and preexponential factor, respectively. $E_{a}$ is the activation energy for diffusion, $k_{B}$ is the Boltzmann constant (approximately $1.3806\times 10^{-23}\;J/K$), and T is the absolute temperature.

According to classical transition state theory, a functional relationship exists between the diffusion coefficient and jump frequency. Wert and Zener et al. [34] established the expression for the atomic jump frequency (Γ) as follows:

(3) $$\Gamma =v_{0}\exp\left(-\frac{E_{a}}{k_{B}T}\right)$$

where $v_{0}$ denotes the vibrational frequency of the interstitial atom. Since the Boltzmann constant is defined as $k_{B}=R/N_{A}$ and the jump frequency follows the Arrhenius equation, the diffusion coefficient can be formulated through the atomic jump frequency as follows:

(4) $$D=L^{2}v_{0}\exp\left(-\frac{N_{A}E_{a}}{RT}\right)$$

where L represents the jump distance, $N_{A}$ is Avogadro’s number (approximately $6.0221\times 10^{23}{\;\mathrm{mol}}^{-1}$), and R denotes the ideal gas constant (approximately $8.3141\;J\cdot {\mathrm{mol}}^{-1}\cdot K^{-1}$). According to the Wert–Zener theory [34], the vibrational frequency $v_{0}$ is given by:

(5) $$v_{0}=\sqrt{E_{a}/2mL^{2}}$$

where m is the atomic mass. The atomic mass of hydrogen used in calculations in this paper is approximately $1.661\times 10^{-27}\;\mathrm{kg}$.

## 3. Results and Discussion

### 3.1. Hydrogen Diffusion Mechanisms in Defect-Free Fe Crystal Lattices

The introduction of hydrogen atoms into metallic materials facilitates the nucleation and propagation of hydrogen-assisted cracking through the free diffusion of hydrogen within the crystal lattice. Consequently, the mechanisms underlying hydrogen-induced embrittlement are intrinsically linked to the diffusion behavior of hydrogen atoms. The hydrogen process behavior in metals is predominantly governed by two critical factors: the preferential occupancy sites of hydrogen within the metal lattice and the energy barriers associated with hydrogen migration.

#### 3.1.1. Preferred Occupation Sites of Hydrogen Atoms

**Figure 2:**
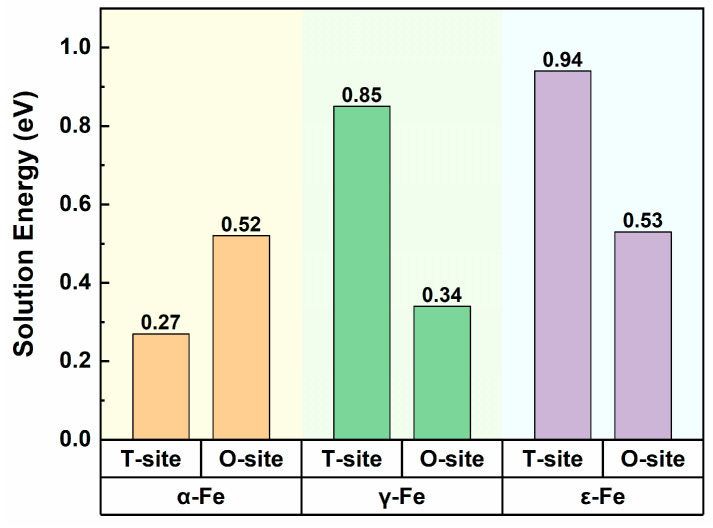
Solution energies of hydrogen atoms at different interstitial sites in α-Fe, γ-Fe, and ε-Fe crystals.

Owing to its small atomic radius, the hydrogen atom typically occupies interstitial sites within the lattice structure of metallic materials and diffuses therein. This diffusion behavior is mediated primarily by the migration of hydrogen atoms between adjacent interstitial sites. Interstitial sites are unoccupied voids within the crystal lattice of metals, typically comprising two types: tetrahedral and octahedral sites. To determine the preferential occupancy sites of hydrogen atoms in different Fe crystals, hydrogen atoms were systematically introduced at both tetrahedral and octahedral interstitial sites in α-Fe, γ-Fe, and ε-Fe crystals. Following comprehensive structural relaxation, the solution energies of the hydrogen atoms at each interstitial site were calculated according to Equation (1), and the results are presented in Figure 2. As observed from the figure, the solution energies of the hydrogen atoms in all the crystals (α-Fe, γ-Fe, and ε-Fe) are positive, indicating endothermic solution processes. This thermodynamic behavior suggests that the hydrogen solution in these crystals is nonspontaneous and requires energy input. Specifically, in α-Fe crystals, the solution energy for octahedral interstitial sites exceeds that of tetrahedral sites, with higher energy values correlated with greater solution difficulty and reduced structural stability of the α-Fe-H system. Consequently, hydrogen atoms are more stable at tetrahedral sites in α-Fe. In contrast, for γ-Fe and ε-Fe, hydrogen atoms have lower energies at octahedral sites than at tetrahedral sites, suggesting preferential hydrogen occupancy at octahedral interstitial positions. These computational results collectively reveal that hydrogen preferentially occupies (i) tetrahedral sites (T-sites) in α-Fe and (ii) octahedral sites (O-sites) in both γ-Fe and ε-Fe. Furthermore, analysis of the lattice parameters revealed that the hydrogen solution induces lattice distortion in the iron matrix, with the degree of distortion dependent on the specific interstitial site occupied by hydrogen [35].

**Figure 3:**
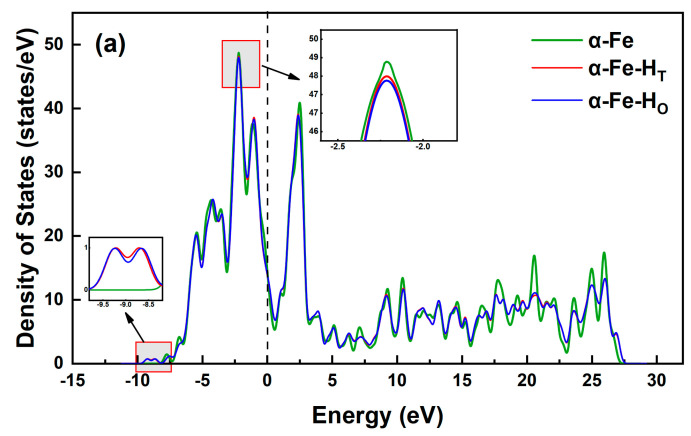

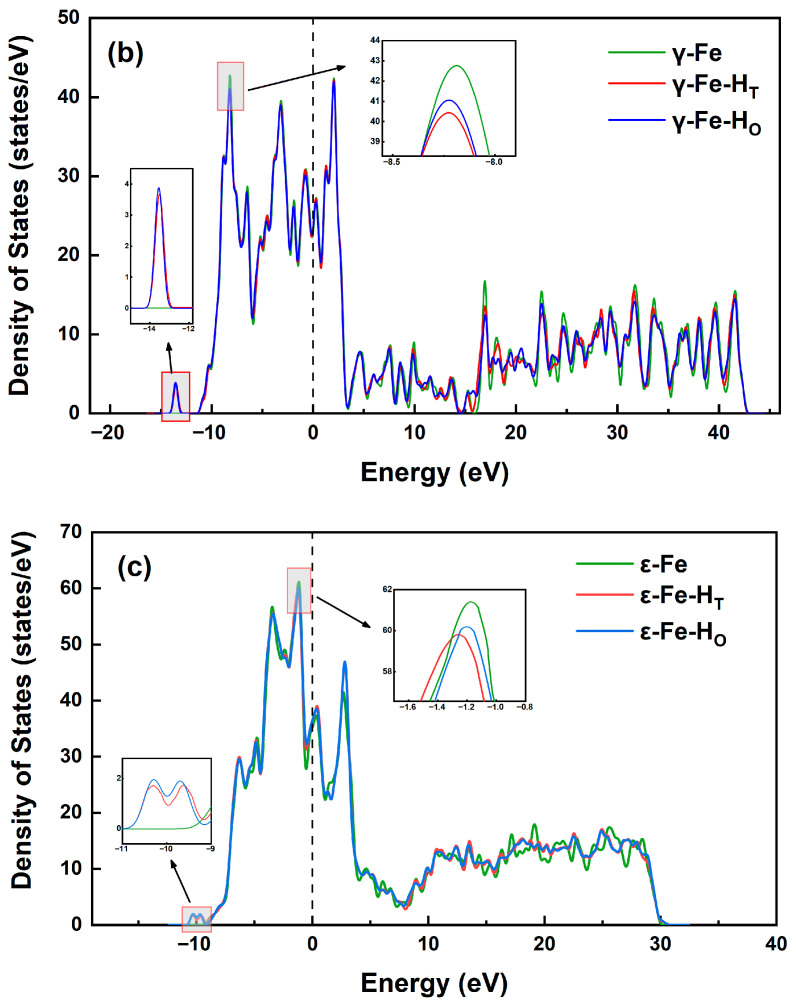
Diagrams of the total density of states of different systems. (**a**) α-Fe; (**b**) γ-Fe; (**c**) ε-Fe.

To further investigate the influence of preferential hydrogen occupancy in Fe crystals, the electronic interactions between hydrogen atoms at different interstitial sites and Fe crystals were examined from the perspective of electronic structure. The total density of states (TDOS) was calculated for α-Fe, γ-Fe, and ε-Fe crystals with hydrogen occupying both tetrahedral and octahedral interstitial sites, as shown in Figure 3. Notably, all the systems exhibit a nonzero density of states at the Fermi level, confirming their metallic character. This electronic feature persists regardless of the interstitial position of the hydrogen, suggesting that the incorporation of hydrogen preserves the metallic nature of the host Fe lattice.

The data in Figure 3a reveal distinct electronic features upon hydrogen incorporation. The magnified view shows that near −2.2 eV, the DOS peak intensity follows the order α-Fe > α-Fe-H_T_ > α-Fe-H_O_. This phenomenon arises because the hydrogen solution induces a partial shift in the electronic states from −2.2 eV to −9.0 eV, where they hybridize with the hydrogen 1s orbitals. Notably, at −9.0 eV, the α-Fe-H_T_ system has a slightly higher peak intensity than does α-Fe-H_O_, indicating greater electron density around the hydrogen in tetrahedral sites. This enhanced electron density suggests stronger chemical bonding when hydrogen occupies tetrahedral sites, providing additional theoretical evidence for preferential tetrahedral site occupancy in α-Fe crystals.

Similarly, the data in Figure 3b demonstrate that in γ-Fe systems, the DOS peak intensity at −8.2 eV decreases in the order of γ-Fe > γ-Fe-H_O_ > γ-Fe-H_T_. Conversely, at −13.5 eV, the peak intensity of the γ-Fe-H_O_ system is marginally greater than that of γ-Fe-H_T_. These observations reveal that hydrogen atoms in octahedral sites exhibit greater electron density and form more stable bonds in γ-Fe, which is consistent with their preferential occupancy of octahedral sites. Parallel conclusions can be drawn for the ε-Fe system on the basis of analogous electronic structure analysis.

**Figure 4:**
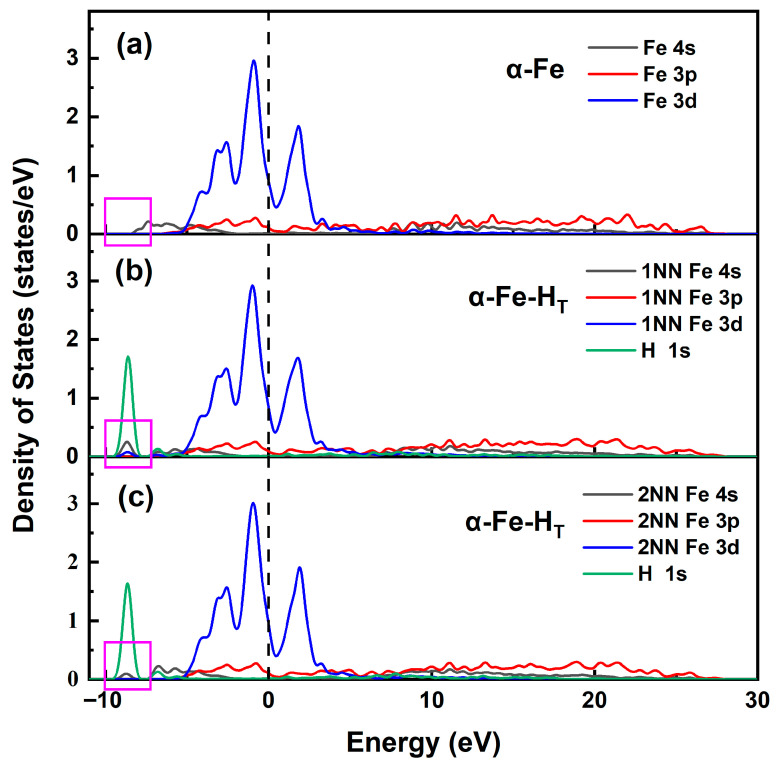
Partial density of states diagrams of different systems. (**a**) α-Fe; (**b**) hydrogen atom at the first nearest-neighbor site relative to the Fe of the α-Fe-H_T_ system (1NN); (**c**) hydrogen atom at the second nearest-neighbor site relative to the Fe of the α-Fe-H_T_ system (2NN).

Figure 4 presents the partial density of states (PDOS) for the α-Fe system and the hydrogen-containing α-Fe-H_T_ system, showing the electronic interactions between hydrogen and its first-nearest-neighbor (1NN) and second-nearest-neighbor (2NN) Fe atoms. The results reveal that the electronic states near the Fermi level are predominantly contributed by Fe 3d orbitals. Upon hydrogen incorporation into the α-Fe interstitial sites, new hybridized peaks emerge in the energy range of −9.5 to −7.8 eV below the Fermi level, originating primarily from H 1s orbitals. These peaks modify the PDOS of neighboring iron atoms, with distinct resonance features appearing between Fe 3d, 4s and H 1s orbitals, indicating the formation of weak Fe-H bonds through slight hybridized conjugation effects. Comparative analysis of the rectangular regions in Figure 4b,c reveals that the PDOS peak intensity of 2NN Fe atoms is significantly weaker than that of 1NN Fe atoms. This attenuation demonstrates the localized nature of the influence of hydrogen, where the electronic perturbation decreases with increasing distance from the hydrogen atom.

#### 3.1.2. Transition State of Hydrogen Diffusion in the Fe Crystal Lattice

**Figure 5:**
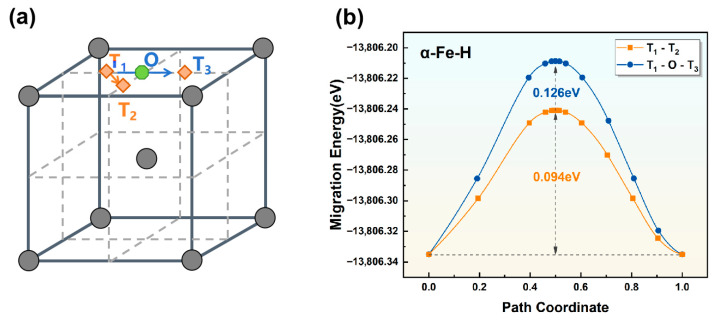
(**a**) Schematic illustration of hydrogen diffusion pathways and (**b**) diffusion energy barriers for hydrogen atoms in α-Fe along the (i) T_1_-T_2_ and (ii) T_1_-O-T_3_ pathways.

**Figure 6:**
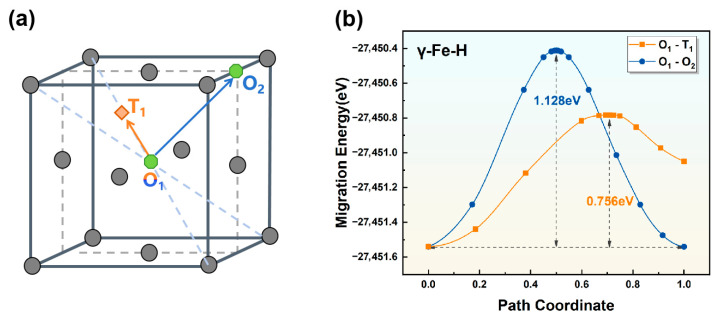
(**a**) Schematic illustration of hydrogen diffusion pathways and (**b**) diffusion energy barriers for hydrogen atoms in γ-Fe along the (i) O_1_-T_1_ and (ii) O_1_-O_2_ pathways.

**Figure 7:**
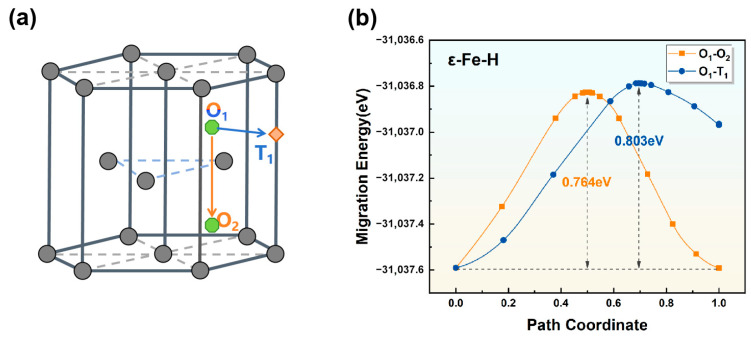
(**a**) Schematic illustration of hydrogen diffusion pathways and (**b**) diffusion energy barriers for hydrogen atoms in ε-Fe along the (i) O_1_-O_2_ and (ii) O_1_-T_1_ pathways.

Previous studies have shown that upon entering the crystal, hydrogen atoms diffuse between two nearest-neighbor stable interstitial sites, governed by the diffusion activation energy. Calculations have identified preferential occupancy sites for hydrogen atoms in different Fe crystal lattices. This work investigates possible diffusion pathways for hydrogen atoms across various unit crystals. The computational results are presented in Figure 5, Figure 6 and Figure 7, where the energy difference between the lowest point (initial state, IS) and the highest point (transition state, TS) in the curves represents the diffusion barrier. The energy maximum, referred to as the saddle point (S), corresponds to the transition-state structure of the crystal, which typically occurs at the midpoint of the atomic diffusion pathway.

(1) Transition state of hydrogen diffusion in the α-Fe crystal lattice

In the α-Fe crystal, hydrogen atoms preferentially occupy tetrahedral interstitial sites (T). This suggests a probable diffusion pathway between adjacent tetrahedral sites (T-T transition). Two distinct diffusion pathways were investigated: (i) T_1_-T_2_ and (ii) T_1_-T_3_, with the corresponding transition states calculated and presented in Figure 5. Notably, the transition state for path (ii) (T_1_-T_3_ diffusion) coincides with the octahedral interstitial position (O), effectively establishing a T_1_-O-T_3_ diffusion pathway. The calculated diffusion barriers are 0.094 eV for path (i) and 0.126 eV for path (ii). The significantly lower energy barrier (0.094 eV) for the T_1_-T_2_ transition indicates a strong preference for hydrogen diffusion between nearest-neighbor tetrahedral sites in α-Fe. Most studies report hydrogen diffusion barriers in α-Fe of approximately 0.1 eV [14], which is consistent with the high mobility of hydrogen in iron. First-principles calculations by Li et al. [36] yielded a diffusion barrier of 0.063 eV, which is in good agreement with our computed value. Specifically, compared with the experimental results, the T_1_-O-T_3_ pathway in α-Fe has a higher barrier (0.126 eV), whereas the energy barrier of the T_1_-T_2_ path is more consistent with the experimental results. This comparative analysis confirms the preferential diffusion of hydrogen between adjacent tetrahedral sites in α-Fe and validates the rationality of the diffusion pathways predicted in this work.

(2) Transition state of hydrogen diffusion in the γ-Fe crystal lattice

In the γ-Fe crystal, hydrogen atoms preferentially occupy octahedral interstitial sites (O). The potential diffusion pathways include direct migration between nearest-neighbor octahedral sites (O-O) or a three-step process via adjacent tetrahedral sites (O-T-O). The transition-state energy profiles for two distinct diffusion paths, (i) O_1_-T_1_ and (ii) O_1_-O_2_, are shown in Figure 6. The calculated diffusion barriers are 0.756 eV and 1.128 eV for paths (i) and (ii), respectively. This indicates that hydrogen requires only 0.756 eV to migrate from an octahedral site (O) to an adjacent tetrahedral site (T) in γ-Fe. These results demonstrate that hydrogen diffusion in γ-Fe predominantly occurs via the lower-energy O-T-O pathway rather than direct O-O hopping because of the significantly lower activation energy barrier. Chohan et al. [37] employed density functional theory to investigate hydrogen diffusion on γ-Fe (100), (110), and (111) surfaces and reported diffusion barriers of 0.6 eV, 0.5 eV, and 0.7 eV, respectively. Complementary experimental studies by Oriani et al. [38] on hydrogen diffusion in pure γ-Fe yielded an activation energy of 0.5 eV, with both sets of results demonstrating good consistency with the computational findings of the present study.

(3) Transition state of hydrogen diffusion in the ε-Fe crystal lattice

The ε-Fe unit cell contains 6 equivalent octahedral interstitial sites (O) and 12 equivalent tetrahedral sites (T). For hydrogen atoms preferentially occupying octahedral sites (O), two primary diffusion pathways exist: (i) direct migration between adjacent octahedral sites (O-O) and (ii) hopping from octahedral to nearest-neighbor tetrahedral sites (O-T). The diffusion energy barriers for these pathways are 0.764 eV for O_1_-O_2_ migration (path i) and 0.803 eV for O_1_-T_1_ transition (path ii) (Figure 7). Transition state analysis reveals that compared with the 0.803 eV barrier for O-T hopping, hydrogen diffusion in ε-Fe preferentially occurs via the lower-energy O-O pathway, which requires an activation energy of 0.764 eV.

#### 3.1.3. Hydrogen Diffusion Coefficient in the Fe Crystal Lattice

Table 2 summarizes the diffusion barriers (E), jump distances (L), and vibrational frequencies (V) of hydrogen atoms in different Fe unit cells (α-Fe, γ-Fe, and ε-Fe). The vibrational frequencies of different unit cells calculated according to Equation (5) are presented in Table 2. By substituting the calculation parameters in Table 2 into Equation (4), the expressions of the diffusion coefficients of hydrogen atoms in different paths within the α-Fe, γ-Fe, and ε-Fe unit cells as functions of temperature are obtained.

(6) $$D_{\alpha -Fe-T1\text{-}T2}=5.3\times 10^{-4}\exp\left(-\frac{9057.088}{RT}\right)\;\;{\mathrm{cm}}^{2}/s$$

(7) $$D_{\alpha -Fe-T1\text{-}O\text{-}T3}=8.7\times 10^{-4}\exp\left(-\frac{12236.704}{RT}\right)\;\;{\mathrm{cm}}^{2}/s$$

(8) $$D_{\gamma -Fe-O1\text{-}T1}=1.9\times 10^{-3}\exp\left(-\frac{72842.112}{RT}\right)\;\;{\mathrm{cm}}^{2}/s$$

(9) $$D_{\gamma -Fe-O1\text{-}O2}=3.8\times 10^{-3}\exp\left(-\frac{108685.056}{RT}\right)\;\;{\mathrm{cm}}^{2}/s$$

(10) $$D_{\varepsilon -Fe-O1\text{-}O2}=2.9\times 10^{-3}\exp\left(-\frac{73612.928}{RT}\right)\;\;{\mathrm{cm}}^{2}/s$$

(11) $$D_{\varepsilon -Fe-O1\text{-}T1}=2.3\times 10^{-3}\exp\left(-\frac{77370.656}{RT}\right)\;\;{\mathrm{cm}}^{2}/s$$

From the aforementioned expressions, it is evident that hydrogen atoms exhibit distinct diffusion activation energies across different Fe crystal structures. Specifically, in the α-Fe unit cell, the diffusion activation energies along the T_1_-T_2_ and T_1_-O-T_3_ pathways are 9.06 kJ mol^−1^ and 12.24 kJ mol^−1^, respectively. In contrast, the γ-Fe unit cell demonstrates significantly higher activation energies of 72.84 kJ mol^−1^ (O_1_-T_1_ pathway) and 108.69 kJ mol^−1^ (O_1_-O_2_ pathway). Notably, the ε-Fe unit cell has intermediate values of 73.61 kJ mol^−1^ (O_1_-O_2_ pathway) and 77.37 kJ mol^−1^ (O_1_-T_1_ pathway). According to comparative analyses, the T_1_-T_2_ pathway in α-Fe has the lowest diffusion activation energy, indicating thermodynamically favorable hydrogen diffusion. This facilitates the accumulation of hydrogen at lattice defects and microcracks under identical environmental conditions, thereby promoting hydrogen-induced brittle fracture. Conversely, the O_1_-O_2_ pathway in γ-Fe presents the highest activation energy (108.69 kJ mol^−1^), which imposes substantial kinetic barriers to hydrogen diffusion. This thermodynamic hindrance effectively suppresses hydrogen atom mobility, thereby mitigating the risk of hydrogen embrittlement.

**Figure 8:**
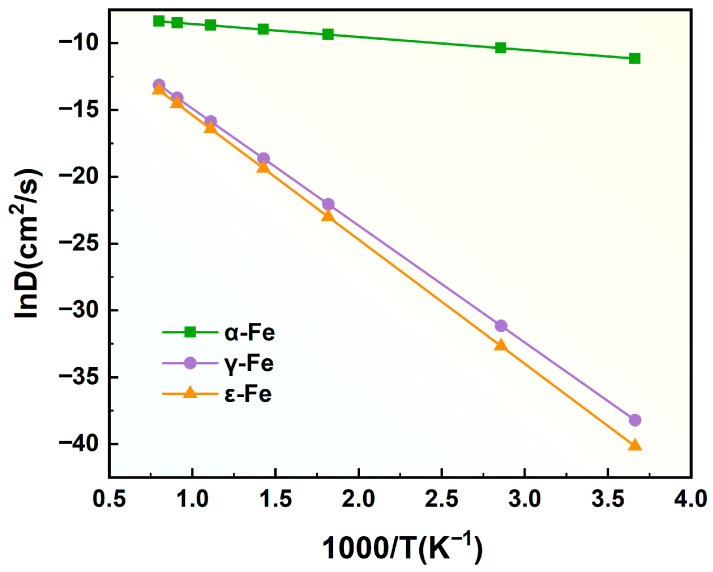
Arrhenius plot of the logarithmic hydrogen diffusion coefficient versus inverse temperature for α-Fe, γ-Fe, and ε-Fe crystals.

To directly compare the hydrogen diffusion behavior in the α-Fe, γ-Fe, and ε-Fe crystal lattices, we systematically calculated the diffusion coefficients of the hydrogen atoms along the T_1_-T_3_ pathway in α-Fe, the O_1_-T_1_ pathway in γ-Fe, and the O_1_-O_2_ pathway in ε-Fe within the temperature range of 273 K to 1273 K. The resulting Arrhenius plots depicting the logarithmic relationship between the diffusion coefficients and reciprocal temperature are presented in Figure 8. These plots demonstrate a linear correlation between the hydrogen diffusion coefficients and temperature reciprocals, with simultaneous observations showing increased diffusion coefficients across all crystal lattices as the temperature increases. Notably, the temperature-dependent convergence of diffusion coefficients among different iron allotropes suggests that elevated thermal energy intensifies atomic vibrations within crystal lattices, facilitating the ability of hydrogen atoms to overcome energy barriers more readily through reduced activation energy requirements. The T_1_-T_3_ pathway in α-Fe exhibited the highest diffusion coefficient because of its relatively low activation energy barrier for hydrogen migration. In contrast, the lower diffusion coefficients observed in the ε-Fe crystal lattices than in the α-Fe and γ-Fe counterparts indicate that materials with ε-martensitic phase structures possess inherent resistance to hydrogen embrittlement. This phenomenon can be attributed to the unique atomic configuration of ε-Fe, which creates higher energy barriers for hydrogen diffusion, thereby suppressing the accumulation of hydrogen at defect sites and typically promoting embrittlement mechanisms.

Hydrogen diffusion coefficients play a critical role in determining the susceptibility of metallic materials to brittle fracture. Yokobori et al. [39] developed a physical model to investigate the relationship between hydrogen embrittlement susceptibility and the interplay of diffusion coefficients with yield strength in metallic systems. Their results demonstrated a direct correlation between the hydrogen embrittlement susceptibility and the hydrogen diffusion rate, where higher diffusion coefficients significantly increase the propensity for hydrogen embrittlement. Shivanyuk et al. [40] conducted pioneering research on the hydrogen-induced γ → ε phase transformation in AISI 310 austenitic steel, revealing that silicon alloying in Cr25Ni20 steel systems reduces the susceptibility to hydrogen embrittlement from 65% to 39%. This protective effect arises from the ability of silicon to increase the quantity of ε-martensite, thereby enhancing the resistance of the material to hydrogen embrittlement through microstructural optimization. Furthermore, Koyama et al. [41] experimentally demonstrated that while hydrogen diffusion behavior remains relatively unchanged during the γ-Fe to ε-Fe martensitic phase transformation, significant hydrogen desorption occurs during the γ-Fe to α-Fe transformation, providing experimental evidence that hydrogen atoms diffuse most rapidly in α-Fe. This computational analysis confirms this hierarchical diffusion behavior, yielding hydrogen diffusion coefficients in the sequence α-Fe > γ-Fe > ε-Fe, which aligns with the aforementioned literature reports.

### 3.2. Interatomic Interactions of Hydrogen in the α-Fe Crystal Lattice

To further investigate the mechanism of hydrogen atoms within the Fe crystal, we systematically investigated the interaction between two hydrogen atoms in the α-Fe crystal. Hydrogen pairs were introduced into tetrahedral interstitial sites at varying separation distances (1.01–3.51 Å), followed by full structural relaxation. Following full structural relaxation, the total energies of each configuration were computed to derive the hydrogen-hydrogen interaction energy ($E_{H}$). A negative interaction energy indicates an attractive force between the hydrogen atoms, whereas a positive value suggests a repulsive interaction [42]. The interaction energy was computed using the following equation:

(12) $$E_{H-H}=\frac{1}{2}\left[E_{FeH_{2}}-E_{Fe}\right]-E_{H-atom}-E_{H}$$

(13) $$E_{H}=E_{FeH}-E_{Fe}-E_{H-atom}$$

where $E_{FeH_{2}}$ denotes the total energy of the α-Fe crystal containing two hydrogen atoms, $E_{Fe}$ is the total energy of the pristine α-Fe crystal, $E_{H-atom}$ is the energy of the hydrogen atoms in their free state, and $E_{H}$ is the hydrogen binding energy.

**Figure 9:**
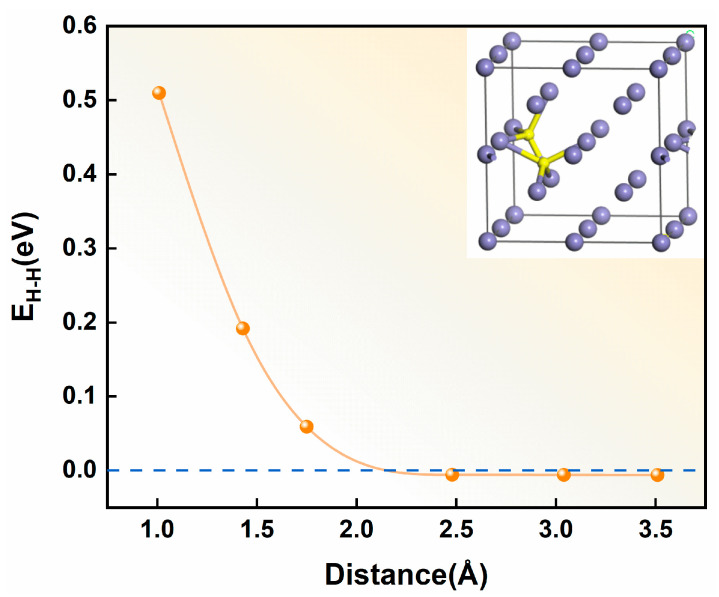
Interaction energy versus interatomic distance for hydrogen atoms in defect-free α-Fe crystals. (In the model schematic, purple spheres represent Fe atoms and yellow spheres represent hydrogen atoms.)

The calculated interaction energy between hydrogen atoms as a function of interatomic distance is shown in Figure 9. The results reveal a distinct distance-dependent interaction behavior: At separation distances below 2.5 Å, hydrogen atoms exhibit positive interaction energies, indicating strong repulsive forces that intensify with decreasing interatomic spacing. This repulsive behavior effectively prevents hydrogen clustering at short ranges. Conversely, when the separation exceeds 2.5 Å, the interaction energy becomes weakly negative (approximately −0.005 eV) and asymptotically approaches zero. This defines a characteristic “screening radius” of 2.5 Å [43], beyond which H–H interactions become negligible. Owing to these repulsive interactions, the hydrogen atoms in α-Fe preferentially occupy interstitial sites separated by distances exceeding the shielding radius. Notably, the equilibrium H-H bond length in molecular hydrogen (0.74 Å) is substantially smaller than this shielding radius. These findings demonstrate that spontaneous hydrogen aggregation and H_2_ molecule formation face significant energetic barriers in defect-free α-Fe crystals, as the required atomic proximity for molecular formation is strongly disfavored by the intrinsic repulsive interactions between hydrogen atoms in the metallic matrix.

On the basis of these findings, it can be reasonably hypothesized that hydrogen aggregation and bubble formation in α-Fe are likely mediated by lattice defects such as vacancies. The accumulation of hydrogen atoms may induce local stress fields and promote vacancy formation. To investigate this possibility, two hydrogen atoms were positioned at varying separation distances within the tetrahedral interstitial sites of a vacancy-containing α-Fe crystal. The hydrogen-hydrogen interaction energy in this defective system was then calculated using the following expression:

(14) $$E_{H-H}=E_{FeH_{2}-V}-2E_{FeH-V}+E_{Fe-V}$$

where $E_{FeH_{2}-V}$ and $E_{FeH-V}$ represent the total energies of vacancy-containing α-Fe crystals with one and two hydrogen atoms, respectively, and $E_{Fe-V}$ corresponds to the total energy of the pristine vacancy-containing α-Fe crystals.

**Figure 10:**
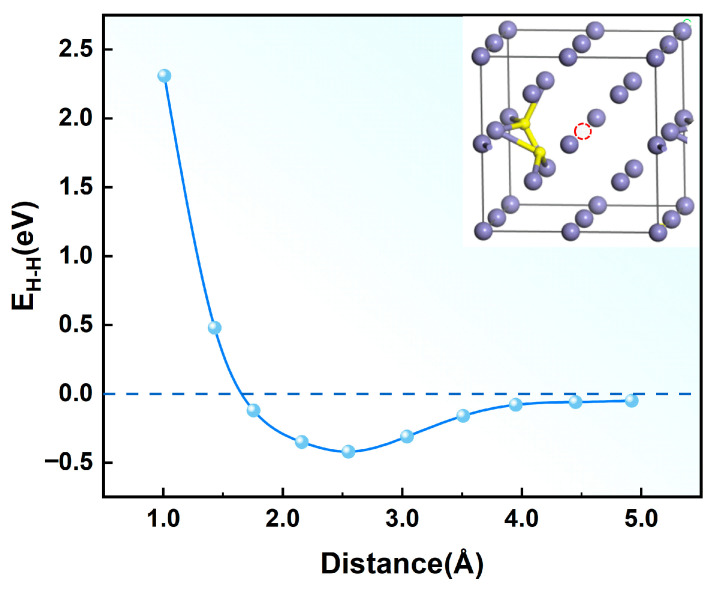
Interaction energy between two hydrogen atoms as a function of interatomic distance in a vacancy-containing α-Fe crystal. (In the model schematic, purple spheres represent Fe atoms, yellow spheres represent hydrogen atoms, and the red dashed line indicates the vacancy site.)

The interaction energy between two hydrogen atoms as a function of the interatomic distance in vacancy-containing α-Fe is shown in Figure 10, revealing complex distance-dependent behavior. At separations below 1.5 Å, hydrogen atoms exhibit strong repulsive interactions that intensify with decreasing distance. Beyond this threshold, the interaction becomes attractive, reaching a maximum strength at 2.5 Å before gradually weakening and approaching zero at larger separations. These findings confirm that vacancy defects substantially enhance the attractive hydrogen-hydrogen interactions in α-Fe, supporting the initial hypothesis. When the number of hydrogen atoms near a vacancy defect reaches a certain threshold, the defect enhances the mutual attraction between hydrogen atoms, leading to the formation of hydrogen molecules. The aggregation and growth of these hydrogen molecules eventually result in the formation of hydrogen bubbles, inducing hydrogen embrittlement in metals. This process aligns with the universal vacancy trapping mechanism reported by Qin et al. [44], wherein hydrogen atoms first saturate the inner surface of the vacancy to form a shielding layer. This layer effectively screens interactions between subsequently trapped hydrogen atoms and surrounding metal atoms, ultimately enabling H_2_ formation at the vacancy core. Moreover, Liu et al. [45] employed first-principles calculations to investigate the interaction between hydrogen atoms and vacancies in W unit cells. They reported that vacancies reduce the surrounding charge density, thereby creating essential prerequisites for hydrogen aggregation and bubble formation. When the hydrogen density within the vacancy reaches a critical threshold, the hydrogen molecules coalesce.

### 3.3. Hydrogen Diffusion Mechanisms in Vacancy-Containing Fe Crystal Lattices

The calculated hydrogen-hydrogen interaction energies in the α-Fe crystal reveal that vacancy defects significantly promote hydrogen accumulation, thereby facilitating hydrogen embrittlement. Building on these findings, this study systematically investigates the influence of vacancy defects on hydrogen diffusion behavior in Fe crystal lattices.

#### 3.3.1. Preferred Occupation Sites of Hydrogen Atoms in Vacancy-Containing Fe Crystal Lattices

**Figure 11:**
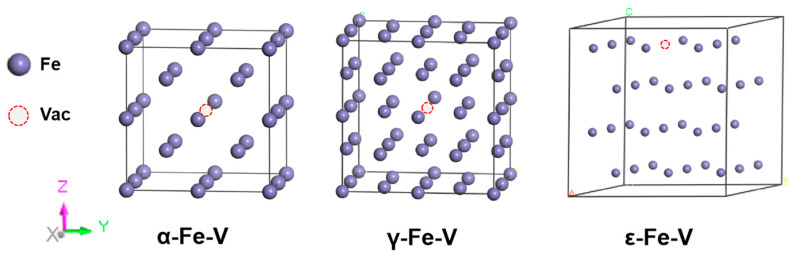
Schematic atomic models of the three vacancy-containing Fe crystal configurations.

Vacancies are among the most prevalent point defects in crystalline materials. The hydrogen vacancy theory proposed by Takai et al. [46] posits that vacancies, rather than the hydrogen atoms themselves, play a dominant role in hydrogen embrittlement, with hydrogen atoms primarily functioning to facilitate vacancy formation. To investigate this phenomenon, in this study, 2 × 2 × 2 supercells of α-Fe, γ-Fe, and ε-Fe crystals were constructed, each containing a monovacancy (Figure 11). The vacancy formation energies were systematically calculated for these systems, as this parameter serves as a critical indicator of vacancy stability within the crystal lattice. The vacancy formation energy ($E_{f}^{V}$) is defined as follows [47]:

(15) $$E_{f}^{V}=E_{Fe_{n-1}V}-\frac{n-1}{n}E_{Fe_{n}}$$

where $E_{Fe_{n-1}V}$ and $E_{Fe_{n}}$ denote the total energies of the supercell systems with and without a monovacancy, respectively. The variable n represents the number of iron atoms in the pristine supercell. The calculated vacancy formation energies for α-Fe, γ-Fe, and ε-Fe crystals according to Equation (15) are 2.15 eV, 4.62 eV, and 5.28 eV, respectively. The calculated vacancy formation energy in α-Fe is in close agreement with Ohnuma’s [48] calculated value of 2.17 eV and matches the experimental value of 2.0 ± 0.2 eV [49]. Remarkably, the introduction of hydrogen atoms at interstitial sites near the vacancy reduces these formation energies to 1.53 eV, 3.22 eV, and 4.62 eV. This significant decrease demonstrates that the presence of hydrogen facilitates vacancy formation, while the resulting vacancies readily trap additional hydrogen atoms, establishing a synergistic interaction between the hydrogen and the vacancies in the iron lattice system.

The calculations confirm that in defect-free α-Fe, γ-Fe, and ε-Fe lattices, hydrogen atoms preferentially occupy tetrahedral interstitial sites, octahedral interstitial sites, and octahedral interstitial sites, respectively. Qiu et al. [50] combined experimental characterization with first-principles calculations to investigate the hydrogen diffusion behavior in 12Cr2Mo1R(H) steel, revealing that vacancy defects significantly alter hydrogen occupation preferences. Building upon these findings, the present study systematically examines how vacancy incorporation affects the preferential occupancy of hydrogen atoms in Fe crystal lattices. To investigate the influence of vacancy defects on hydrogen site preference, hydrogen atoms were introduced at both tetrahedral and octahedral interstitial sites near the vacancy in vacancy-containing α-Fe. Geometric optimization results demonstrate a significant modification of the hydrogen occupation behavior near vacancies. Hydrogen atoms no longer occupy tetrahedral sites and instead spontaneously migrate toward and stabilize at distorted octahedral sites adjacent to the vacancy. These preferred positions are characterized by slight displacements from ideal octahedral centers, shifted toward the vacancy yet not coinciding with the exact geometric center of the interstitial site. In vacancy-containing γ-Fe, hydrogen atoms initially placed at octahedral interstitial sites spontaneously migrate toward adjacent tetrahedral sites during structural optimization, stabilizing at positions shifted approximately 1.27 Å from the vacancy center. Similarly, hydrogen atoms originating at tetrahedral sites relocate to positions approximately 1.21 Å from the vacancy center. An analogous behavior is observed in ε-Fe with vacancies: Hydrogen atoms initially at octahedral sites are displaced by 0.61 Å toward the vacancy (final position ≈ 1.1 Å from center), whereas those starting at tetrahedral sites move 0.5 Å (final position ≈ 0.97 Å from center). These systematic observations demonstrate that in both γ-Fe and ε-Fe containing vacancies, hydrogen atoms stably occupy positions around octahedral or tetrahedral interstitial sites near the vacancies rather than at the centers of the interstitial sites.

**Figure 12:**
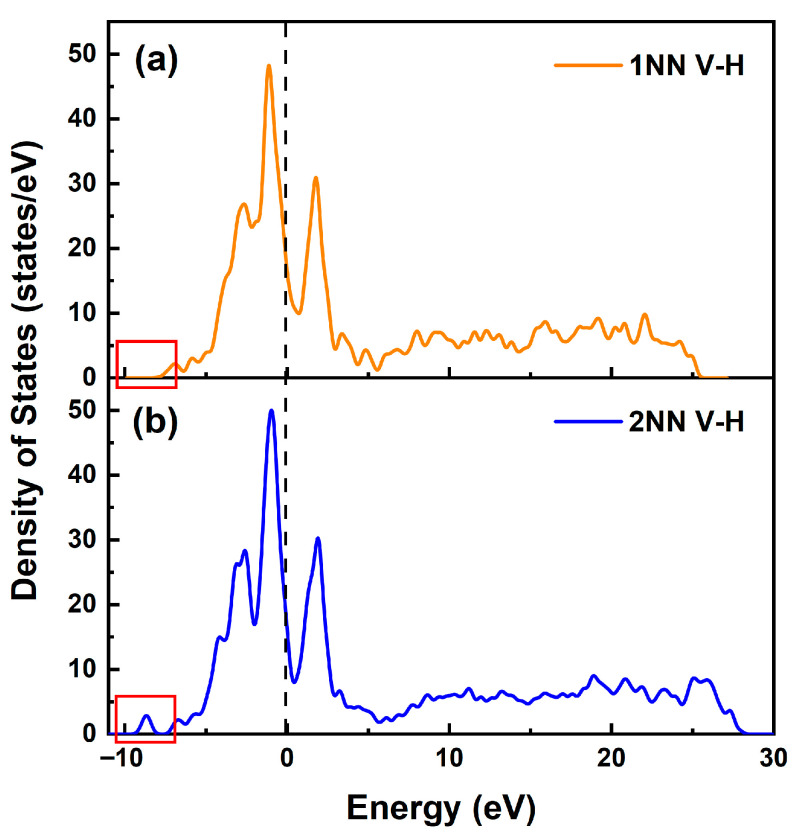
The total density of hydrogen in vacancy-containing α-Fe crystals. (**a**) Hydrogen atom at the first nearest-neighbor site relative to the vacancy (1NN); (**b**) hydrogen atom at the second nearest-neighbor site relative to the vacancy (2NN).

As shown in Figure 12, the total density of states was calculated for hydrogen atoms occupying both the first-nearest-neighbor (1NN V-H) and second-nearest-neighbor (2NN V-H) positions relative to the vacancy in the α-Fe crystal, providing atomic-scale insights into vacancy-mediated hydrogen site preference. As shown in Figure 3a, after the hydrogen atom enters the α-Fe crystal, a new hybridization peak appears between −9.5 eV and −8.0 eV below the Fermi level. This is because there is a slight hybridization conjugation effect between the iron atom and the hydrogen atom, and the hydrogen atom will form an Fe-H bond with the nearest iron atom. Notably, the DOS profile for hydrogen at the 1NN position (Figure 12a, red rectangle) shows complete suppression of these characteristic hybridization peaks within the −9.5 to −8.0 eV range, demonstrating full hydrogen capture by the vacancy without Fe-H bonding. When hydrogen occupies the 2NN positions (Figure 12b), the reappearance of hybridization peaks confirms the highly localized nature of vacancy effects, with perturbed electronic interactions extending only to immediate atomic neighbors.

#### 3.3.2. Transition State of Hydrogen Diffusion in a Vacancy-Containing Fe Crystal Lattice

**Figure 13:**
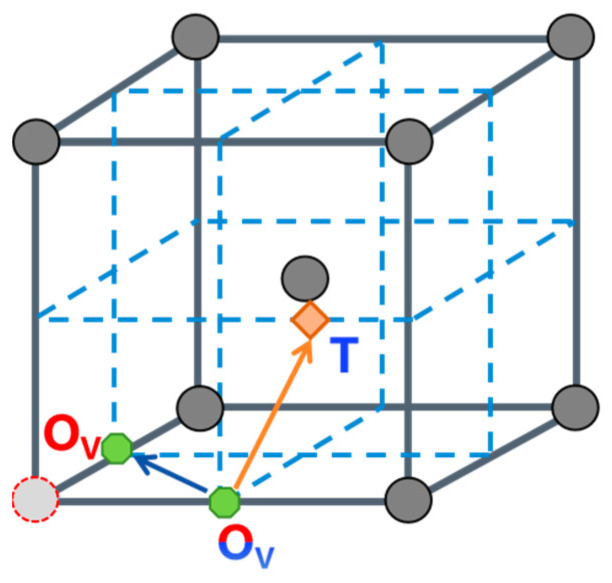
Schematic diagrams of hydrogen diffusion paths in vacancy-containing α-Fe crystals.

**Figure 14:**
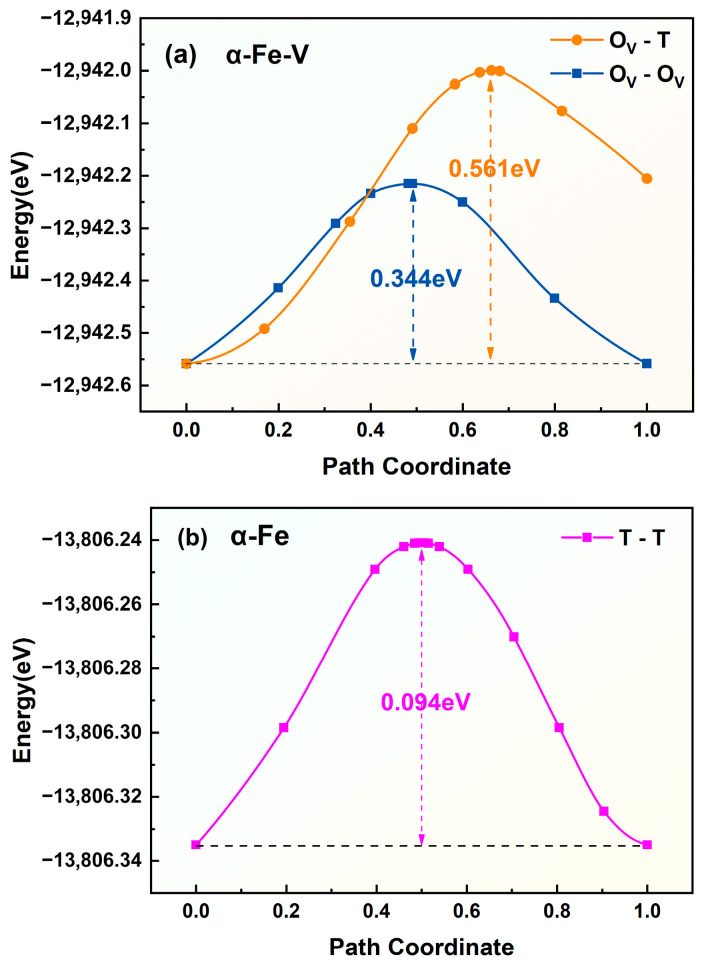
Diffusion energy barriers for hydrogen migration in different crystal systems. (**a**) Vacancy-containing α-Fe crystal (α-Fe-V); (**b**) α-Fe crystal.

In vacancy-containing α-Fe, hydrogen atoms preferentially occupy octahedral interstitial sites adjacent to the vacancy (denoted as O_V_ sites). For a trapped hydrogen atom to escape the vacancy, it must first diffuse to a more distant tetrahedral interstitial site (T site) before proceeding through the conventional T-T diffusion pathway in pristine α-Fe. This suggests a likely escape route involving migration from a vacancy-proximal octahedral site to a vacancy-distal tetrahedral site (O_V_-T pathway). Furthermore, the diffusion energy barrier was calculated for hydrogen migration from a vacancy-proximal octahedral site to a vacancy-proximal tetrahedral site (O_V_-T_V_ pathway), with the corresponding diffusion paths illustrated schematically in Figure 13. The diffusion energy barriers for hydrogen migration in vacancy-containing α-Fe are shown in Figure 14a, revealing distinct energy landscapes for different diffusion pathways. The barrier of the O_V_-T path is 0.561 eV, whereas that of the O_V_-O_V_ pathway is 0.344 eV. This substantial difference (0.217 eV) demonstrates the strong trapping effect of vacancies on hydrogen atoms, as the system preferentially undergoes lower-energy O_V_-O_V_ diffusion rather than the more energetically demanding O_V_-T transition. The 0.561 eV barrier for O_V_-T migration represents the critical energy threshold for hydrogen to escape vacancy trapping and subsequently diffuse through perfect α-Fe via the T-T pathway, which requires only 0.094 eV, as shown in Figure 14b. These results quantitatively establish that vacancies create a local energy minimum of 0.217 eV for hydrogen atoms, effectively serving as trapping sites while simultaneously inhibiting hydrogen release into the bulk lattice.

**Figure 15:**
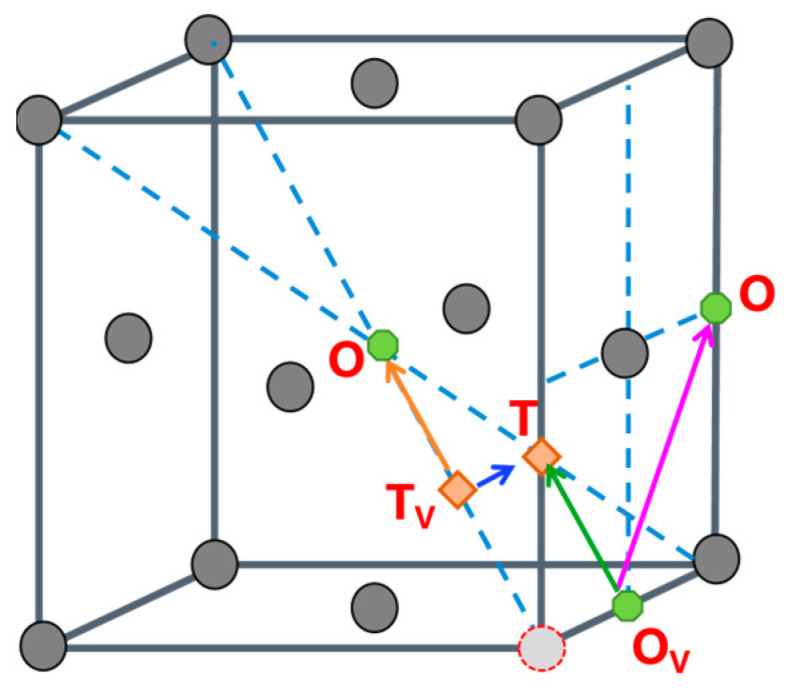
Schematic diagrams of hydrogen diffusion paths in vacancy-containing γ-Fe crystals.

**Figure 16:**
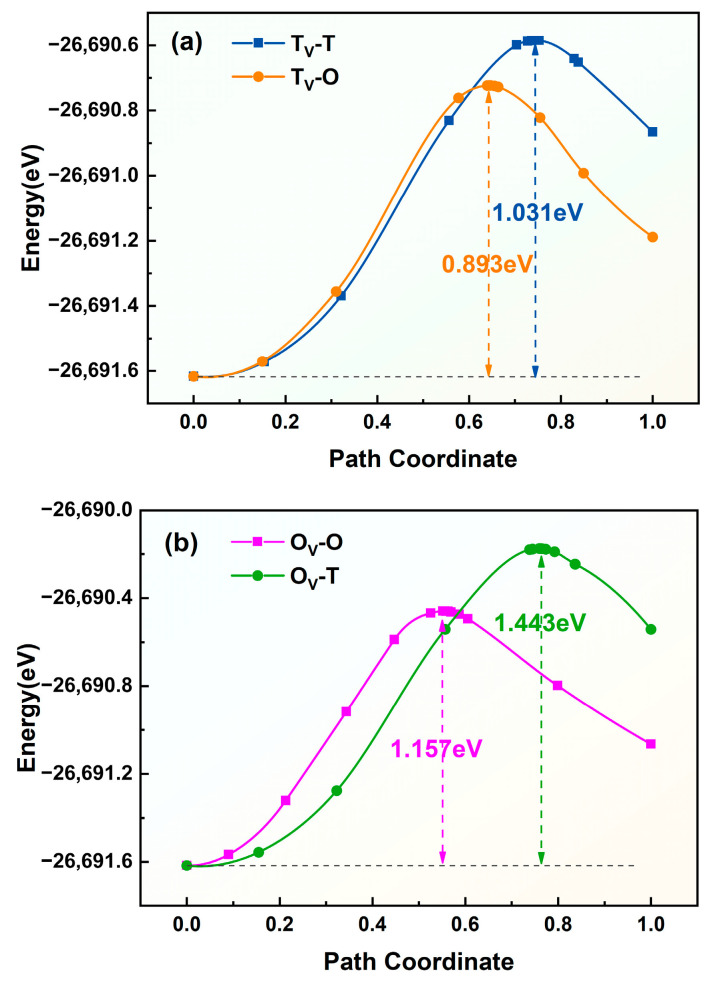
Diffusion energy barriers for various pathways in vacancy-containing γ-Fe (γ-Fe-V). (**a**) T_V_-T and T_V_-O pathways; (**b**) O_V_-O and O_V_-T pathways.

In vacancy-containing γ-Fe, hydrogen atoms preferentially stabilize near octahedral (O) or tetrahedral (T) interstitial sites adjacent to the vacancy. To overcome the trapping effect of vacancies on hydrogen atoms, four possible diffusion pathways exist, labeled O_V_-T, O_V_-O, T_V_-T, and T_V_-O, whose schematic representations are shown in Figure 15. The diffusion energy barriers for hydrogen migration along these pathways near the vacancy are illustrated in Figure 16 The barriers for the T_V_-T, T_V_-O, O_V_-O, and O_V_-T pathways are 1.031 eV, 0.893 eV, 1.157 eV, and 1.443 eV, respectively. This finding indicates that hydrogen atoms escaping from vacancy defects preferentially diffuse via the T_V_-O pathway.

**Figure 17:**
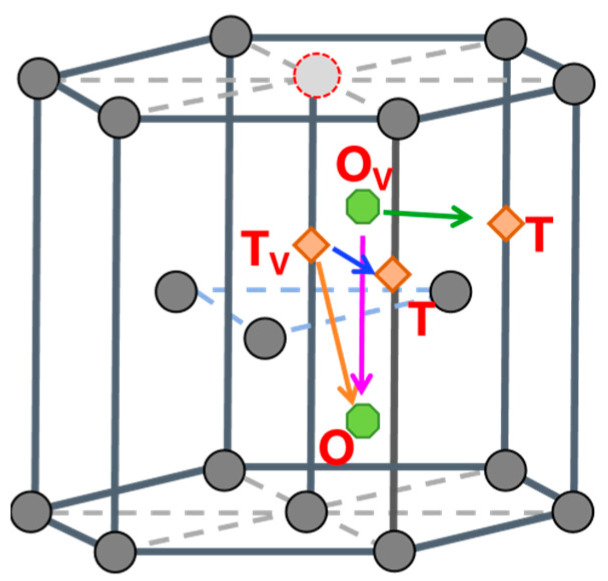
Schematic diagrams of hydrogen diffusion paths in vacancy-containing ε-Fe crystals.

**Figure 18:**
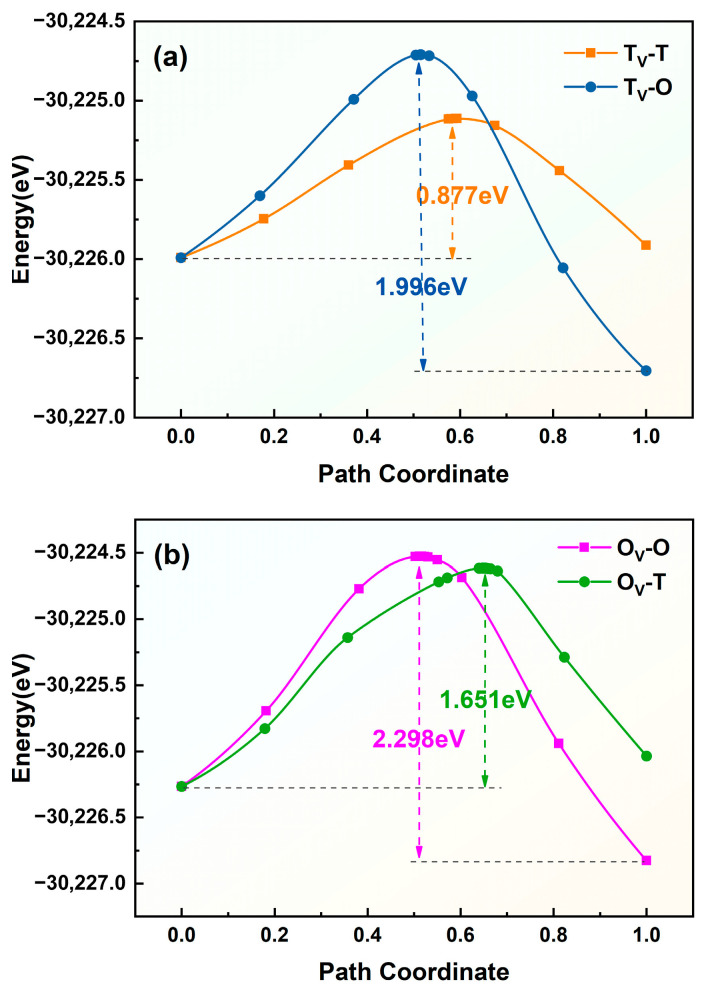
Diffusion energy barriers for various pathways in vacancy-containing ε-Fe (ε-Fe-V). (**a**) T_V_-T and T_V_-O pathways; (**b**) O_V_-O and O_V_-T pathways.

Similarly, hydrogen atoms preferentially occupy the octahedral or tetrahedral interstitial sites near the vacancy in the vacancy-containing ε-Fe crystal. Consequently, there are four possible diffusion pathways, designated T_V_-T, T_V_-O, O_V_-O, and O_V_-T, as illustrated in Figure 17. The diffusion energy barriers for hydrogen atoms migrating along these different paths near the vacancy are shown in Figure 18. The barriers for diffusion along the T_V_-T, T_V_-O, O_V_-O, and O_V_-T paths are 0.877 eV, 1.996 eV, 2.298 eV, and 1.651 eV, respectively. This finding indicates that within the ε-Fe crystal containing a vacancy, hydrogen atoms tend to diffuse preferentially along the T_V_-T pathway. Interestingly, hydrogen atoms must overcome a higher diffusion energy barrier to break free from the confinement of vacancies and diffuse into defect-free crystal lattices than is required for diffusion within crystal lattices. 

## 4. Conclusions

In this study, first-principles calculations are employed to systematically investigate the hydrogen diffusion mechanisms in both perfect and vacancy-containing Fe crystal lattices (α-Fe, γ-Fe, and ε-Fe). The computational analysis determines the preferential occupancy sites and diffusion pathways of hydrogen in these crystalline systems, with diffusion coefficients derived using the Arrhenius equation. Furthermore, the influence of vacancy defects on both hydrogen-hydrogen interactions and hydrogen diffusion behavior in Fe crystalline systems is thoroughly investigated. The main conclusions can be obtained as follows:

(1) Analysis of the solution energies and electronic density of states reveals distinct hydrogen occupancy preferences across Fe crystal lattices: Hydrogen atoms preferentially occupy tetrahedral interstitial sites (T) in α-Fe crystals but preferentially occupy octahedral sites (O) in both γ-Fe and ε-Fe crystals.
(2) In the α-Fe crystal, hydrogen preferentially diffuses via the T-T pathway with an energy barrier of 0.094 eV and an activation energy of 9.057 kJ/mol. For the γ-Fe crystal, overcoming a substantially higher barrier of 0.756 eV and an activation energy of 72.842 kJ/mol is needed. In the ε-Fe crystal, hydrogen primarily migrates through the O-O pathway with a 0.764 eV barrier and an activation energy of 73.612 kJ/mol. These results demonstrate that hydrogen diffusion is most facile in α-Fe, as evidenced by its significantly lower energy barrier and correspondingly higher diffusion coefficient than those of the other crystalline phases.
(3) When the distance between two hydrogen atoms in α-Fe exceeds 2.5 Å, their interaction energy is very low. When the distance is less than 2.5 Å, a significant repulsive force develops between the hydrogen atoms, making it challenging for them to aggregate and form hydrogen gas within intact, defect-free α-Fe. However, the introduction of vacancy defects enhances the attractive forces between hydrogen atoms, thereby facilitating the formation of hydrogen bubbles. Furthermore, vacancy defects alter the preferential occupation sites and diffusion pathways of hydrogen atoms in Fe crystals. Instead of residing at the interstitial centers, hydrogen atoms are positioned at locations slightly shifted toward the vacancies. Additionally, hydrogen atoms must overcome higher diffusion energy barriers to escape the trapping effect of vacancies and diffuse into the defect-free crystal.

## Figures and Tables

**Table 1:** The lattice constant of α-Fe, γ-Fe and ε-Fe.

	Space Group	Present	Theory	Experiment
α-Fe	Im-3m	a = b = c = 2.851 Å	a = b = c = 2.836 Å [25] a = b = c = 2.869 Å [26]	a = b = c = 2.866 Å [27]
γ-Fe	Fm-3m	a = b = c = 3.456 Å	a = b = c = 3.44 Å [28] a = b = c = 3.56 Å [24]	a = b = c = 3.56 Å [29]
ε-Fe	P63/mmc	a = b = 2.46 Å, c/a = 1.581	a = b = 2.45 Å, c/a = 1.585 [30]	a = b = 2.46 Å, c/a = 1.598 [31]

**Table 2:** Diffusion barriers (E), jump distances (L), vibrational frequencies ($v_{0}$) and diffusion activation energy ($E_{a}$) of hydrogen atoms in α-Fe, γ-Fe, and ε-Fe crystals.

Unit Cell Type	Diffusion Path	Diffusion Barrier E (eV)	Jump Distance L (Å)	Vibrational Frequency $v_{0}\;(s^{-1})$	Diffusion Activation Energy $E_{a}\;(\mathrm{kJ}/\mathrm{mol})$
α-Fe	Pathway (i): T_1_-T_2_	0.094	1.43	$0.43\times 10^{13}$	9.06
	Pathway (ii): T_1_-O-T_3_	0.126	1.01	$0.52\times 10^{13}$	12.24
γ-Fe	Pathway (i): O_1_-T_1_	0.756	2.03	$0.91\times 10^{13}$	72.84
	Pathway (ii): O_1_-O_2_	1.128	1.24	$1.21\times 10^{13}$	108.69
ε-Fe	Pathway (i): O_1_-O_2_	0.764	1.94	$0.78\times 10^{13}$	73.61
	Pathway (ii): O_1_-T_1_	0.803	1.49	$1.04\times 10^{13}$	77.37

## Data Availability

The original contributions presented in this study are included in the article. Further inquiries can be directed to the corresponding authors.
